# Terpinen-4-ol inhibits the proliferation and mobility of pancreatic cancer cells by downregulating Rho-associated coiled-coil containing protein kinase 2

**DOI:** 10.1080/21655979.2022.2054205

**Published:** 2022-03-24

**Authors:** Wenpeng Cao, Ruhua Tian, Runsang Pan, Baofei Sun, Chaolun Xiao, Yunhua Chen, Zhirui Zeng, Shan Lei

**Affiliations:** a Department of Anatomy, School of Basic Medicine, Guizhou Medical University, Guiyang, China; b Department of Physiology, School of Basic Medicine, Guizhou Medical University, Guiyang, China; c Department of Pathophysiology, School of Basic Medicine, Guizhou Medical University, Guiyang, China

**Keywords:** Pancreatic cancer, terpinen-4-ol, ROCK2, proliferation, mobility

## Abstract

Terpinen-4-ol (T4O), a compound isolated from the seeds of turmeric, has exhibited anti-malignancy, anti-aging, and anti-inflammatory properties in previous studies. However, the specific effects and molecular mechanisms of T4O on pancreatic cancer (PC) cells remain largely unknown. In this study, we demonstrated that T4O markedly suppressed PC cell proliferation and colony formation *in vitro* and induced apoptosis. Similarly, T4O significantly inhibited the migration and invasion of PC cells *in vitro*. Through RNA sequencing, 858 differentially expressed genes (DEGs) were identified, which were enriched in the Rhodopsin (RHO)/ Ras homolog family member A (RHOA) signaling pathway. Rho-associated coiled-coil containing protein kinase 2 (ROCK2), a DEG enriched in the RHO/RHOA signaling pathway, was considered as a key target of T4O in PC cells; it was significantly reduced after T4O treatment, highly expressed in PC tissues, and negatively associated with patient outcome. Overexpression of ROCK2 significantly reduced the inhibitory effects of T4O on PC cell proliferation and mobility. Moreover, T4O inhibited cell proliferation *in vivo* and decreased the Ki-67, cell nuclear antigen, EMT markers, and ROCK2 expression. In conclusion, we consider that T4O can suppress the malignant biological behavior of PC by reducing the expression of ROCK2, thus contributing to PC therapy.

## Graphical abstract

**Figure:**
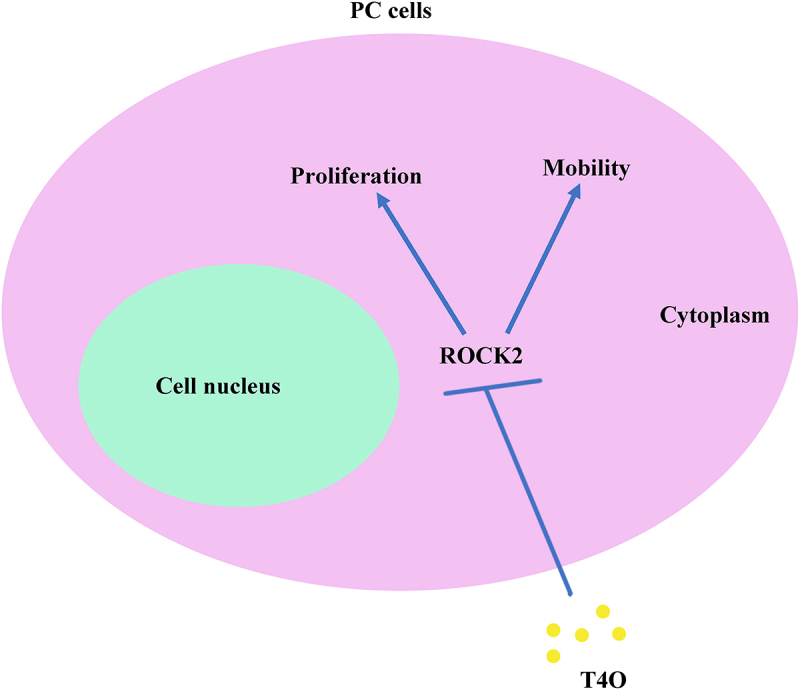

## Introduction

Pancreatic cancer is a malignant tumor with poor prognosis and remains difficult to diagnose and treat [1]. Although considerable effort has been made in molecular understanding and disease therapy, morbidity and mortality rates are still elevated, with a 5-year survival rate of < 1% [2,3]. Furthermore, due to its low response rate to radio- or chemotherapy as well as lack of effective biomarkers, therapeutic success still remains low [4,5]. Therefore, there is an urgent need to explore novel therapeutic drugs and targets for PC.

Terpinen-4-ol (T4O) is an active substance of the turmeric species that has exhibited anti-tumor, anti-aging, and anti-inflammatory properties in previous studies [6,7]. Ken *et al*. reported that T4O inhibits colorectal cancer growth by increasing the levels of reactive oxygen species [8]. Wu *et al*. revealed that T4O induces apoptosis in human non-small cell lung cancer [9]. However, the effects of T4O and its underlying mechanisms on PC have not yet been clarified.

Rho-associated coiled-coil containing protein kinase 2 (ROCK2) is one of the most important effector downstream molecules of the Rho subfamily [10]. It has been found that ROCK2 protein plays an important role in the occurrence and development of tumors, mainly participating in the regulation of tumor cell survival and apoptosis as well as the invasion and metastasis of malignant tumors [11]. Researchers have reported that ROCK family proteins are upregulated in tumors and mediate the malignant biological behavior of cancer [12,13]. Rath *et al*. reported that ROCK2 promoted PC cell invasion and metastasis [14]. Hu *et al*. confirmed that mi-R4435-2HG promotes the progression of ovarian carcinoma by upregulating ROCK2 [15]. To date, some inhibitors, including KD025 and HA-1077 targeting ROCK2, have been identified and have exhibited distinct anti-tumor effects [16,17]. Therefore, ROCK inhibition could be considered as a chemotherapeutic target for pancreatic cancer.

In the present study, we aimed to identify the specific effects and key molecular mechanisms of T4O in PC, which may aid in the treatment of PC. It has been demonstrated that T4O treatment causes ROCK2 downregulation and induces cell apoptosis and EMT repression, thus leading to the inhibition of PC cell proliferation and migration. This evidence reveals a novel mechanism of T4O in anti-PC activity, thus providing a promising strategy for PC therapy.

## Materials and methods

### Collection of PC tissues

PC and corresponding adjacent tissues (n = 25) were collected from the Affiliated Hospital of Guizhou Medical University, with the approval and supervision of the Human Ethics Committee of Guizhou Medical University (approval number: 2022–10). PC tissues were collected prior to receiving chemo- and radiotherapy; all patients provided written informed consent. The PC tissues were stored at −80°C before the experiments.

### Cell culture, medicine, and transient transfection

Two PC cell lines (AsPC-1 and PANC-1) were purchased from the ATCC. Both cell lines were cultured in DMEM (Gibco, USA) with 10% fetal bovine serum (FBS; BI, Israel) at 37°C with 5% CO_2_. T4O was purchased from Wuhan Fengyao Tonghui Chemical Products Co. Ltd. ROCK2 overexpression and empty vector plasmids were purchased from GeneCopoeia (Guangzhou, China). For transient transfection, PC cells were seeded in 6-well plates, whereas plasmids were transfected into cells using Lipofectamine 2000 (Invitrogen, Carlsbad, CA, USA) until cells reached 50% confluence. The transfection efficiency in PC cells was further determined via western blotting, followed by incubation at 37°C with 5% CO_2_ for 48 h.

### Cell count kit-8 (CCK-8)

AsPC-1 and PANC-1 cells (3 × 103) were plated into 96-plate wells. Different concentrations (0, 0.5, 1, and 2 μM) of T4O were used to treat the cells. Each treatment group was set up in six repetitive wells. After 24 and 48 h, 10 μl CCK-8 reagent (Invitrogen, USA) was added to each well. The proliferative rates of cells were determined using a spectrophotometer at 450 nm. The experiments were performed in triplicate.

### Colony formation assay

A total of 5 × 10^3^ PC cells were placed in six-well plates. Different concentrations (0, 0.5, 1, and 2 μM) of T4O were used to treat the cells. Each treatment group consisted of three replicates. After culturing for 10 days, the cell colonies were immobilized with 4% paraformaldehyde and stained with 0.5% crystal violet (Boster, Wuhan, China). The experiments were performed in triplicate.

### Flow cytometry analysis

After 24 h, PC cells were seeded in six-well plates and treated with different concentrations (0, 0.5, 1, and 2 μM) of T4O. After washing three times with PBS, the cells were stained using the Annexin V-FITC Apoptosis Detection Kit (Boster, Wuhan, China) and then detected using a DeFLEX flow cytometer (Beckman, USA). The results were analyzed using Flwo JO.

### Western blot assay

Total protein in the PC cells was extracted using radio-immunoassay precipitation lysis buffer (Beyotime Biotechnology, Suzhou, China) containing phenylmethylsulfonyl fluoride (Servicebio, Wuhan, China). The protein concentrations of the samples were determined using a bicinchoninic acid (BCA) protein assay (Servicebio, Wuhan, China). The 10% sodium dodecyl sulfate polyacrylamide gels (Meilune, Dalian, China) were employed in separating proteins, which were then transferred to polyvinylidene fluoride membranes (Thermo Scientific, USA).

After blocking the membranes with skimmed milk powder (Beyotime Biotechnology, Suzhou, China), they were incubated with primary antibodies containing ROCK2 (dilution 1:1000; Cat no A2395; ABclonal, China), Bcl2 (dilution 1:1000; Cat no A19693; ABclonal, China), pro-caspase3 (dilution 1:1000; Cat no A11953; ABclonal, China), cleave-caspase3 (dilution 1:500; Cat no A11021; ABclonal, China), N-cadherin (dilution 1:1000; Cat no 22,018-1-AP; Proteintech, China), E-cadherin (dilution 1:1000; Cat no 20,874-1-AP; Proteintech, China), vimentin (dilution 1:1000; Cat no 10,366-1-AP; Proteintech, China), and GAPDH (dilution 1:2000; Cat no 10,494-1-AP; ProteinTech, China) for 16 h at 4°C. After washing twice with Tris-buffered saline containing 0.1% Tween‐20, the membranes were incubated with secondary antibody and then visualized using an enhanced chemiluminescence reagent. GAPDH was used as the loading control to calculate relative protein expression.

### Wound healing assay

PC cells were placed in six-well plates. A 200-μL tip was used to create a wound in the monolayer cell. The cells were washed twice with PBS to remove the floating cells and replace the fresh medium. T4O (0, 0.5, 1, and 2 μM) was used to culture the PC cells. Wound healing conditions were recorded from 0 to 24 h using an optical microscope.

### Transwell assay

PC cells (1 × 10^5^ PC cells/well) were resuspended in 150 μl FBS-free DMEM and added to the upper transwell chambers (ThermoFisher Scientific, USA). The upper chambers were pre-coated with Matrigel (ThermoFisher Scientific, USA), while the lower chambers were set with 700 μl DMEM medium containing 10% FBS. Different concentrations (0, 0.5, 1, and 2 μM) of T4O were used to treat PC cells. After 24 h, the invading cells were immobilized and stained with 0.5% crystal violet. Finally, an inverted microscope was used to photograph the invasive cells in the chambers; the number of cells in five random fields in each chamber was counted.

### RNA sequencing and bioinformatics analysis

Total RNA was extracted from PANC-1 cells treated with DMSO and 2 μM T4O for 24 h using TRIzol Reagent (Invitrogen; Thermo Fisher Scientific, USA), according to the manufacturer’s instructions. RNA library construction and sample sequencing were conducted by Hangzhou Lianchuan Biological Information Technology Co., Ltd. RNA quality was analyzed using NanoDrop™ 2000c (NanoDrop Technologies; Thermo Fisher Scientific, USA). The Illumina HiSeq 2500 platform was used to sample the sequences. Original reads with greater than 10% unknown beads and of low quality were filtered. The count number was used as a unit to quantify the expression levels of the genes. The differentially expressed genes were analyzed using the EdgeR package, with a cutoff value of |LogFC| > 1 and an adjusted P value of < 0.05. The pathways of differentially expressed genes were analyzed using KOBAS (http://kobas.cbi.pku.edu.cn/anno_iden.php) [18]. Pathways with a P value of < 0.05 was set as significantly enriched.

### qRT-PCR analysis

TRIzol was used to extract RNA from PC tissues and cells, according to the manufacturer’s instructions; RNA purity and concentration were determined using a UV spectrophotometer. RNA was reverse-transcribed into cDNA using the Super Script III First-Strand Synthesis System (Vazyme, Shanghai, China). The relative amount of the target gene was calculated by comparison with the internal control GAPDH. The primers used in this study are detailed as follows:

### Primers used in sample analysis

**Table:** 

GENE	Sequence (5’ -> 3’)	
ARHGEF37	Forward Primer	CTGAGGACAGATCGCTGCTTC
	Reverse Primer	CGGCTCCTGATGTCAGAGG
ARHGEF6	Forward Primer	TCCTCGCTGAAAAATGGGGTA
	Reverse Primer	CTTGGAGGGTTGCACATCCT
MYL9	Forward Primer	TCTTCGCAATGTTTGACCAGT
	Reverse Primer	GTTGAAAGCCTCCTTAAACTCCT
ARHGAP35	Forward Primer	AGTGTGGTGGGATTATCTGGG
	Reverse Primer	CGAAGCGGTTGCACAAACAA
ARHGEF11	Forward Primer	ATGAGTGTAAGGTTACCCCAGAG
	Reverse Primer	CGTTGAACGAGACCTGTTGT
ARHGEF2	Forward Primer	CAGGCATGACCATGTGCTATG
	Reverse Primer	TTTACAGCGGTTGTGGATAGTC
ARHGEF28	Forward Primer	AGGTGATGAAGTCTACGCTAACT
	Reverse Primer	AGTGGCAGTGATTCCCTCTAT
MYL10	Forward Primer	TCGACAAAGAGGACTTGAGGG
	Reverse Primer	ACACCGTGAAGTTGATGGGTC
MYL2	Forward Primer	TTGGGCGAGTGAACGTGAAAA
	Reverse Primer	CCGAACGTAATCAGCCTTCAG
MYL5	Forward Primer	ACACTCATGGATCAGAACCGA
	Reverse Primer	GCGTTAAGAATGGTCTCCTCG
MYL7	Forward Primer	GCCCAACGTGGTTCTTCCAA
	Reverse Primer	CTCCTCCTCTGGGACACTC
RAC3	Forward Primer	TCCCCACCGTTTTTGACAACT
	Reverse Primer	GCACGAACATTCTCGAAGGAG
ROCK2	Forward Primer	TCAGAGGTCTACAGATGAAGGC
	Reverse Primer	CCAGGGGCTATTGGCAAAGG
GAPDH	Forward Primer	GGAGCGAGATCCCTCCAAAAT
	Reverse Primer	GGCTGTTGTCATACTTCTCATGG

### ROCK2 clinical value analysis

The gene expression profile of PC tissues and the corresponding clinical information were obtained from the ICGC database (https://icgc.org/content/icgc-home) [19]. Patients with missing information on overall (OS) and disease-free survival (DFS) were excluded. ROCK2 expression in patients with stages I–II and III–IV was analyzed using an unpaired t-test. Patients with PC were divided into high and low expression groups according to the median expression value of ROCK2; Kaplan-Meier survival analysis was used to analyze the difference in DFS and OS between these two groups. Statistical significance was set at P < 0.05.

### Subcutaneous tumorigenesis model

Animal experiments were approved by the Ethics Committee of Guizhou Medical University and were in accordance with the Chinese Guidelines for Ethical Review of Laboratory Animal Welfare (approval number: 2,200,047). Ten female BALB/c nude mice were purchased from the Animal Center of Guizhou Medical University (Guizhou, China). Following adaptive feeding, 2 × 10^6^ PANC-1 cells were subcutaneously injected into the right upper flank of mice. After injecting the cells for 1 week, mice with tumors reaching 40–60 mm^3^ were randomly divided into DMSO and T4O treatment groups (n = 5 per group). The T4O group was intraperitoneally injected with 40 mg/kg T4O every 5 days, while the DMSO group were injected with an equal volume of DMSO (the control) every 5 days. The health status of the mice was monitored daily, while the tumor volume was measured per week. Tumor volume was calculated using the following formula: (mm^3^) = (Long×Width^2^)/2. All mice were subjected euthanasia after a 5-week treatment; tissues originating from PANC-1 cells were extracted to determine the expression of E-cadherin, N-cadherin, vimentin, KI67, and ROCK2.

### Immumohistochemical staining (IHC)

Paraffin-embedded tumor tissues extracted from mice were immersed in differently graded xylene and ethanol solutions for dewaxing and rehydration. After antigen retrieval using sodium citrate and blocking of endogenous peroxidase activity using H_2_O_2_, the tissue samples were incubated with KI67 (dilution 1:2000; Cat no 27,309-1-AP; Proteintech, Wuhan, China), PCNA (dilution 1:400; Cat no 10,205-2-AP; Proteintech, Wuhan, China), N-cadherin (dilution 1:1000; Cat no 22,018-1-AP; Proteintech, China), E-cadherin (dilution 1:500; Cat no 20,874-1-AP; Proteintech, China), vimentin (dilution 1:2500; Cat no 10,366-1-AP; Proteintech, China), and ROCK2 (dilution 1:250; Cat no 66,633-1-Ig; Proteintech, Wuhan, China) overnight at 4°C. After washing the uncombined antibody with PBS, PC tissues were incubated with the secondary antibody, followed by DAB and hematoxylin staining. Finally, a microscope (Olympus, Tokyo, Japan) was used to collect the images.

### Statistical analysis

All experiments were repeated three times; the results were analyzed using SPSS software (version 19.0). Unpaired t-test and one-way analysis of variance combined with the least significant difference t-test were conducted to determine differences between the two groups as well as in multiple groups, respectively; P < 0.5 was set as the significance threshold.

## Results

T4O significantly suppressed PC cell PANC-1 and AsPC-1 cell proliferation *in vitro* and induced PC cell apoptosis. It also inhibited PC cell mobility *in vitro* and suppressed EMT. A total of 858 DEGs were identified in PANC-1 cells after treatment with 2 μM T4O via RNA-seq. These genes were enriched in the RhoA/Rho kinase signaling pathway, whereas ROCK2 expression decreased most significantly according to the DEGs. Furthermore, a high expression of ROCK2 was observed in PC tissues, as compared to that in the adjacent tissues; this predicted a poor prognosis. ROCK2 overexpression reversed the inhibitory effects of T4O on PC cell proliferation and mobility. T4O suppressed the proliferation of PANC-1 cells *in vivo* and reduced the expression of EMT markers, KI67, PCNA and ROCK2.

### *T4O suppressed PC cell proliferation, induced PC cell apoptosis* in vitro

**Figure 1.**
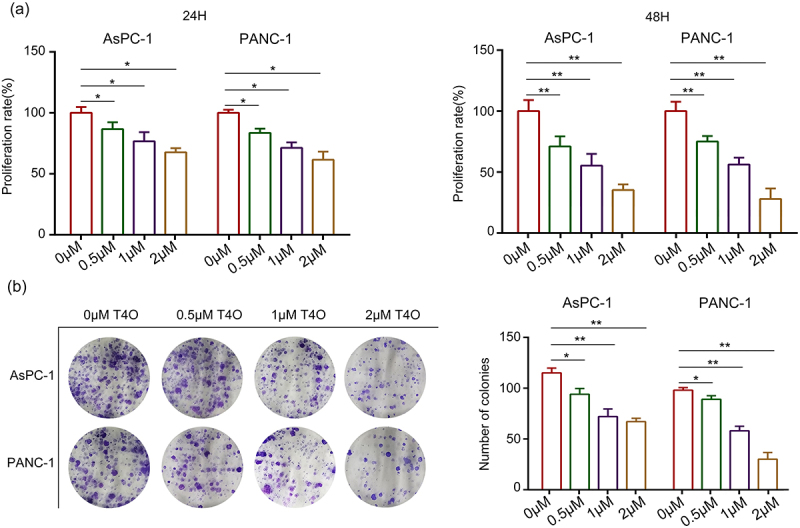
**T4O inhibits PC cell proliferation in vitro**. (a) AsPC-1 and PANC-1 cells were treated with different concentrations (0, 0.5, 1 and 2 μM) of T4O; CCK-8 assay was used to detect the proliferation in each group. (b) Colony formation assay was used to detect the colony formation of PC cells treated with different concentrations (0, 1, 2 and 4 μM) of T4O. *, P < 0.05; **, P < 0.01.

**Figure 2.**
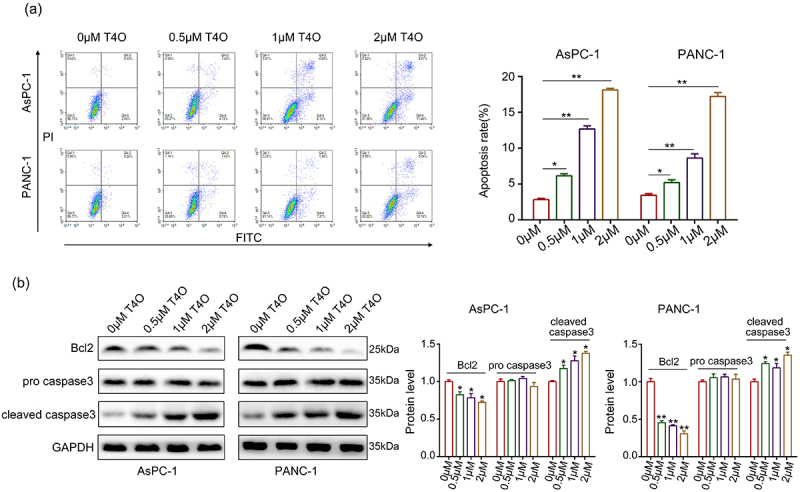
**T4O induces PC cell apoptosis in vitro**. (a) AsPC-1 and PANC-1 cells were treated with different concentrations (0,0.5,1, and 2 μM) of T4O; flow cytometry was used to detect cell distribution in each group. (b) Western blotting was used to detect the expression of AsPC-1 and PANC-1 in cells treated with different concentrations (0,0.5,1, and 2 μM) of T4O. *, P < 0.05; **, P < 0.01.

Different concentrations (0, 0.5, 1, and 2 μM) of T4O were used to treat the PC cells. As a result, CCK-8 assays demonstrated that T4O markedly reduced the proliferation rate of AsPC-1 and PANC-1 cells at both 24 and 48 h (Figure 1a). Furthermore, colony formation assays revealed that PC cells in the T4O treatment group had low colony formation ability (Figure 1b). Flow cytometry analysis demonstrated that AsPC-1 and PANC-1 cells treated with T4O had a significantly increased apoptosis rate (Figure 2a). Western blot results demonstrated that T4O significantly decreased the expression of Bcl-2 in AsPC-1 and PANC-1 cells, as well as increased the expression of cleaved caspase3 (Figure 2b). These results indicate that T4O significantly suppressed the proliferation of PC cells and induced apoptosis.

### *T4O reduced the migration and invasion of PC cells* in vitro

**Figure 3.**
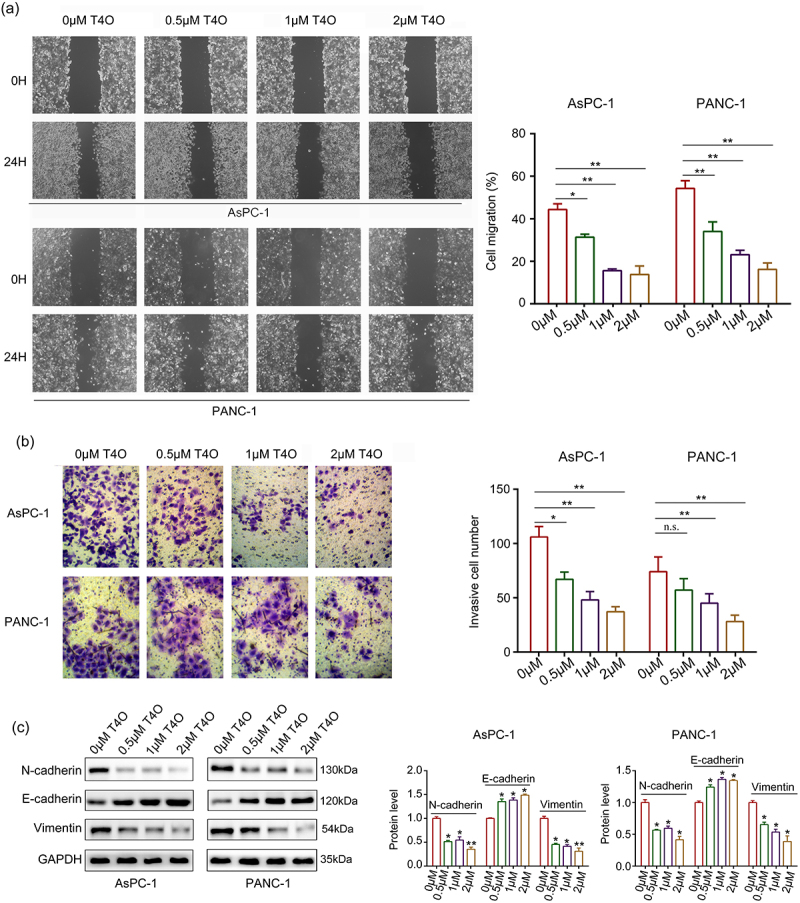
**T4O suppresses the motility of PC cells in vitro**. (a) Wound healing assays were used to determine the migration rate of PC cells treated with different concentrations (0,0.5,1, and 2 μM) of T4O. (b) Transwell assays were used to detect the number of invasive PC cells treated with different concentrations (0,0.5,1, and 2 μM) of T4O. (c) Western blotting was used to detect the expression of E-cadherin, vimentin, and N-cadherin in PC cells treated with T4O. *, P < 0.05; **, P < 0.01.

Wound healing assays were performed; the results indicated that the migration ability of AsPC-1 and PANC-1 cells were all reduced after T4O treatment (Figure 3a). Similarly, T4O treatment markedly reduced the invasive abilities of AsPC-1 and PANC-1 cells (Figure 3b). Furthermore, western blotting indicated that T4O treatment prominently reduced the expression of N-cadherin and vimentin as well as upregulated E-cadherin expression (Figure 3c). Taken together, these results indicated that T4O reduces the motility and EMT phenotype of PC cells.

### ROCK2 is identified as a key target of T4O

**Figure 4.**
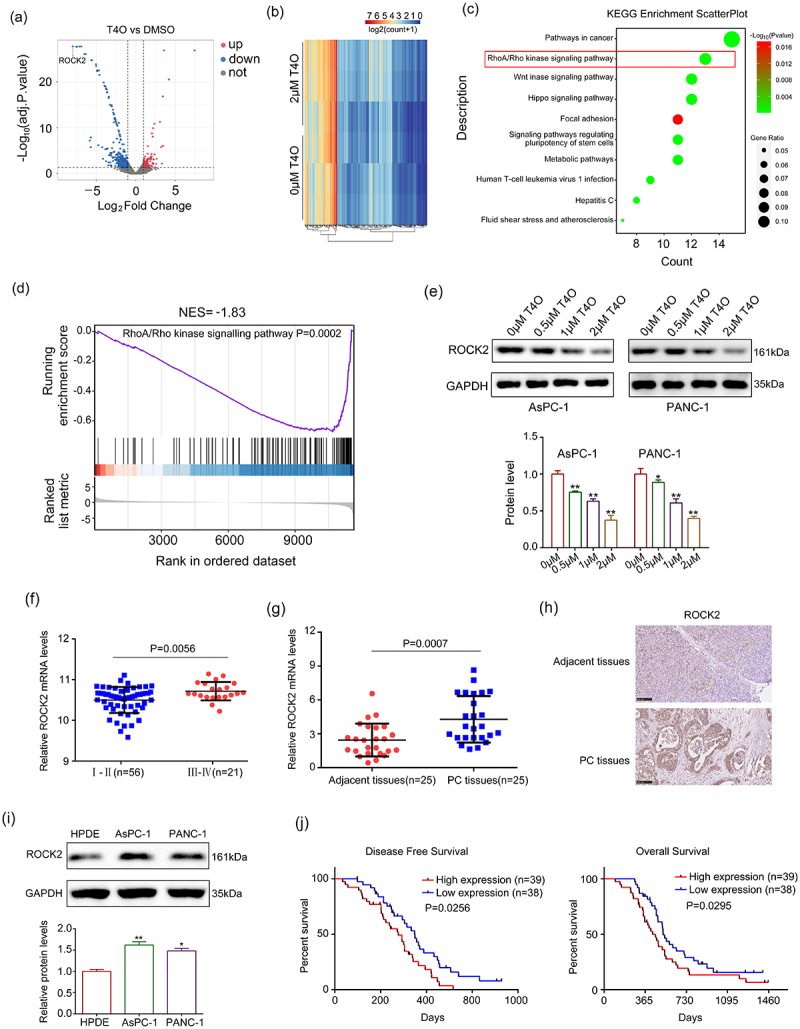
**ROCK2 is identified as a key target of T4O**. (a-b) Differentially expressed genes (DEGs) in the PC cells treated with DMSO and T4O were identified. (c) KEGG analysis was performed to determine the pathways of enriched DEGs. (d) Gene enrichment plots showing a series of genes enriched in the RhoA/Rho kinase signaling pathway. (e) Western blot was used to detect the protein levels of ROCK2 in PC cells treated with different concentrations (0, 0.5, 1 and 2 μM) of T4O. (f) ROCK2 expression in PC tissues from patients in stages I–II and III–IV according to the data from ICGC. (g) qRT-PCR was used to detect the expression of ROCK2 in PC and adjacent tissues. (h) IHC was used to detect the expression of ROCK2 in PC and adjacent tissues. (i) Western blot was used to detect the expression of ROCK2 in HPDE, AsPC-1, and PANC-1 cells. (j) KM plot showing the OS and DFS in patients with low and high ROCK2 expression according to the data from ICGC database. *, P < 0.05; **, P < 0.01.

RNA sequencing was performed to further explore the molecular mechanisms of T4O in PC cells. A total of 362 upregulated genes and 496 downregulated genes with |LogFC | ≥ 1 and adjusted P value of < 0.05 were identified in T4O treatment cells (Figure 4a-b; Supplementary table 1). KEGG analysis revealed that these 858 differentially expressed genes (DEGs) were significantly enriched in cancer, RhoA/Rho kinase signaling, Wnt signaling, Hippo signaling, and metabolic pathways (Figure 4c). Furthermore, by gene set enrichment analysis (GSEA), we found that many RhoA/Rho kinase signaling pathway-related genes were enriched in the T4O treatment group but negatively associated with T4O treatment (Figure 4d). As evidence that activation of the RhoA/Rho kinase signaling pathway is a key factor in PC progression, we focused on this pathway.

Interestingly, among DEGs enriched in the RhoA/Rho kinase signaling pathway, ROCK2 decreased most significantly after 2 μM T4O treatment, according to the RNA-seq and qRT-PCR results (Table 1). Similarly, the protein level of ROCK2 decreased after T4O treatment (Figure 4e). Moreover, according to the data obtained from the ICGC database, we found that the expression of ROCK2 was higher in the PC tissues of patients in stages III–IV, as compared with those of patients in stages I–II (Figure 4f). Furthermore, both ROCK2 mRNA and protein levels were increased in PC tissues from our research group when compared to their corresponding adjacent tissues (Figure 4g-h). Compared to HPDE cells, the expression of ROCK2 in the PC cell lines of AsPC-1 and PANC-1 was elevated (Figure 4i). Furthermore, according to data from the ICGC database, we found that patients with high ROCK2 expression had lower DFS and OS (Figure 4j). Therefore, we considered ROCK2 as a key T4O-regulated gene.

**Table 1.** Change level of differently expressed genes (DEGs) enriched in RHOA/RHO pathway, according to the data from RNA-seq and qRT-PCR (T4O treatment group *vs* DMSO treatment group). *, P < 0.05; **, P < 0.01

ID	RNA-seq (LogFC count+1)	qRT-PCR (relative expression in PANC-1)	qRT-PCR (relative expression in AsPC-1)
ARHGEF37	2.586**	1.334*	1.229*
ARHGEF6	1.503**	1.003	0.989
MYL9	1.521**	1.322*	1.443**
ARHGAP35	−1.281*	0.773*	0.698*
ARHGEF11	−1.114*	0.744*	0.711*
ARHGEF2	−1.047*	0.812*	0.776*
ARHGEF28	−1.863**	0.662*	0.499**
MYL10	−1.040*	0.934	0.898
MYL2	−2.162**	0.668**	0.521**
MYL5	−1.036*	0.942	0.988
MYL7	−1.000*	0.832*	0.722*
RAC3	−1.614**	0.769*	0.712*
ROCK2	−7.976**	0.339**	0.244**

### Overexpression of ROCK2 alleviated the suppressor functions of T4O on PC cell proliferation and migration

**Figure 5.**
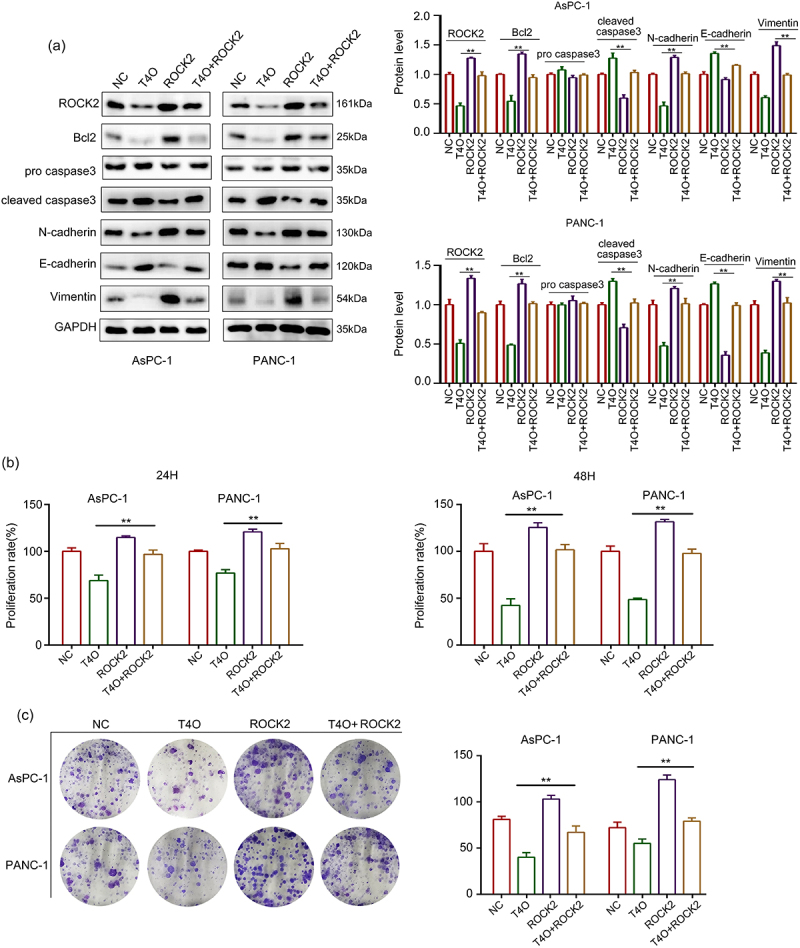
**Overexpression of ROCK2 reversed the inhibitory effects of T4O on proliferation of PC cells**. PC cells were treated with DMSO + NC, T4O + NC, DMSO + ROCK2 plasmid, and T4O + ROCK2 plasmid, respectively. (a) Western blot was used to detect the expression of ROCK2, Bcl2, pro-caspase-3, cleave-caspase-3, N-cadherin, E-cadherin, and Vimentin in each group of PC cells. (b) CCK-8 assay was used to detect the proliferative rate of PC cells in each group in 24 and 48 h. (c) colony formation assays was used to detect the colony formation of PC cells in each group. **, P < 0.01.

**Figure 6.**
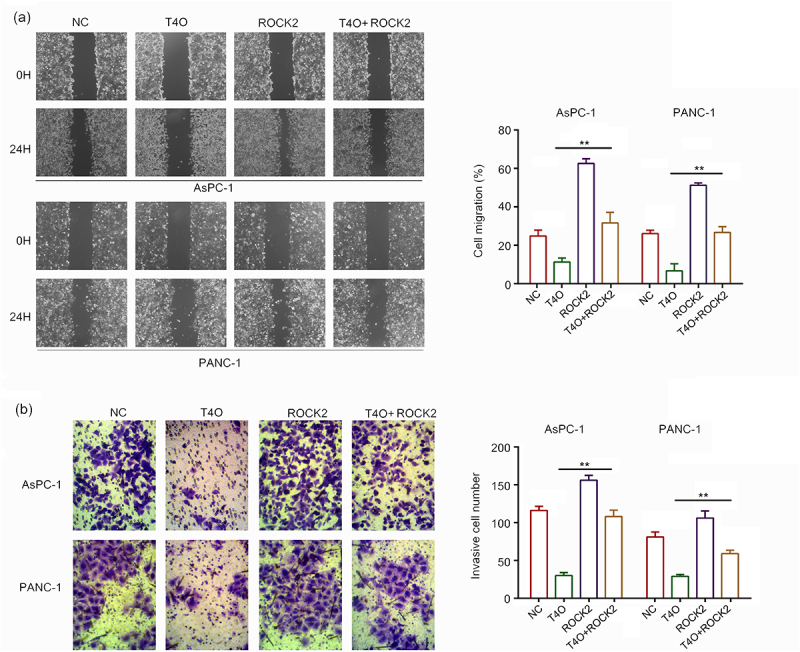
**Overexpression of ROCK2 reversed the inhibitory effects of T4O on migration and invasion PC cells**. PC cells were treated with DMSO + NC, T4O + NC, DMSO + ROCK2, and T4O + ROCK2 plasmids. (a) Wound healing assays were performed to determine the migration rate of the PC cells in each group. (b) Transwell assays were performed to detect the number of invasive PC cells in each group. **, P < 0.01.

It has been widely demonstrated that ROCK2 is an oncogenic gene in a series of cancers; therefore, we hypothesized that ROCK2 may be involved in T4O-induced biological functions. We transfected ROCK2 plasmids into PC cells prior to T4O treatment. The western blot results demonstrated that T4O significantly decreased the expression of Bcl-2, N-cadherin, vimentin and ROCK2 in AsPC-1 and PANC-1 cells, as well as increased the expression of cleaved caspase3 and E-cadherin; overexpression of ROCK2 can reverse these effects (Figure 5a). The results of the CCK-8 assay showed that ROCK2 overexpression alleviated the suppressive effects of T4O on PC cell proliferation at both 24 and 48 h (Figure 5b). The results of the colony formation assays showed that ROCK2 overexpression alleviated the suppressive effects of T4O on the colony formation ability of PC cells (Figure 5c). Wound healing assays indicated that ROCK2 overexpression significantly alleviated the suppressive effects of T4O on the migration potential of PC cells (Figure 6a). Transwell assays indicated that ROCK2 overexpression significantly reversed the inhibitory effects of T4O on the invasive potential of PC cells (Figure 6b). These results indicated that ROCK2 downregulation is involved in the T4O-induced effects in PC.

### *T4O suppressed the proliferation of PC cells* in vivo

**Figure 7.**
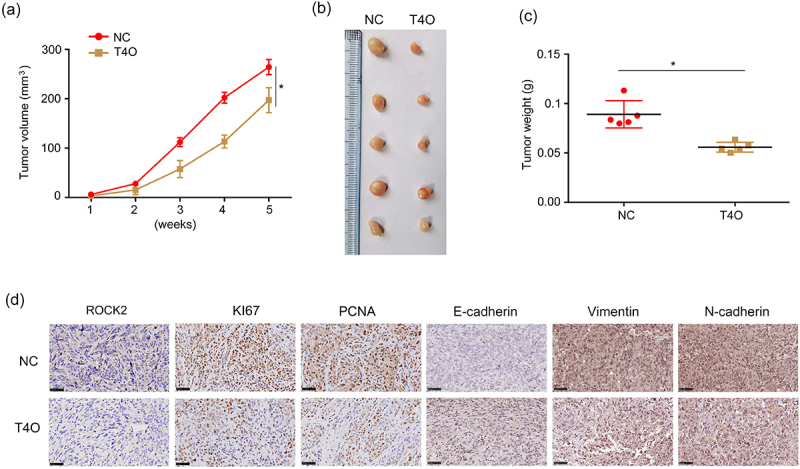
**T4O represses PC cell proliferation in vivo**. (a-b) Tumor volumes of tissues treated with DMSO and T4O. (c) Tumor weights of tissues treated with DMSO or T4O. (d) Expression of ROCK2, E-cadherin, N-cadherin, vimentin, PCNA, and KI67 in tumor tissues treated with DMSO and T4O. **, P < 0.01.

A subcutaneous tumorigenesis model was used to detect the effect of T4O on the proliferation of PC cells *in vivo*. T4O treatment and control PANC-1 cells were subcutaneously injected into the right upper flank of the mice. The results showed that PC tissues treated with T4O grew slower and had lower weights (Figure 7a-c). IHC results showed that PC tissues treated with T4O exhibited lower expression of ROCK2, vimentin, N-cadherin, PCNA, and KI67, as well as increased E-cadherin expression (Figure 7d).

## Discussion

Excision and chemotherapy are the main therapeutic strategies for early PC; however, only a few PC patients benefit from these strategies because of severe side effects and unstoppable recurrence [20,21]. Thus, it is necessary to uncover the mechanisms underlying PC progression and to develop novel therapeutic methods for patients with PC. Because of an increasing body of evidence indicating that some natural products exhibit chemo-preventive potential with low toxicity, a growing interest in the use of natural products as chemo-preventive agents has ensued [^22–24^].

Until now, bioactive molecules derived from natural medicinal plants have been approved by Food and Drug Administration as anti-cancer drugs [25,26]. For example, turmeric, a natural member of the *Zingiberaceae* family, exhibited anti-carcinoma effects in various cancer models via different signaling pathways, including PC [^27–29^]. T4O is a monomer compound [30] that is found in many plant essential oils; it reportedly possesses anti-tumor, anti-inflammatory, and anti-bacterial properties [^31–33^]. A previous study indicated that T4O can be used as an effective natural compound for inhibiting the proliferation of HL-60 cells by inducing autophagy and apoptosis [34]. In the present study, through a series of cell functional experiments, we found that T4O significantly inhibited PC cell proliferation, colony formation, migration, and invasion, as well as induced apoptosis. This evidence demonstrated that T4O exhibits distinct anti-PC effects *in vitro*.

It is widely known that natural products are characterized by their structural diversity; therefore, the molecular mechanisms of natural products are highly complex [35,36]. RNA-seq is a novel technology that objectively evaluates the molecular mechanisms of drugs at the omics level; observing the change of molecule landscape and biological functions after drug treatment is made possible through RNA-seq [37,38]. In this study, RNA-seq was performed to explore the molecular mechanisms underlying T4O. Results revealed that a total of 858 genes were significantly changed after T4O treatment; these DEGs were enriched in the RHOA/RHO pathway. Moreover, we found that ROCK2 expression was decreased most significantly among DEGs that were enriched in the RHOA/RHO pathway. Therefore, ROCK2 has attracted the attention from us.

ROCK2 is a serine/threonine kinase that regulates a series of cellular functions, including cytoskeletal rearrangement, reactive oxygen species production, cell movement, and transcription [39,40]. Previous studies have demonstrated that ROCK2 plays a key role in the progression of PC; its suppression may inhibit cancer progression. Liu et al. showed that ROCK2 promotes the migration and invasion of PC cells [41]. Peng et al. revealed that miR-200b/c targets ROCK2; inhibition of ROCK2 signaling pathways inhibits the proliferation and metastasis of cholangiocarcinoma cancer [42]. These studies highlight that targeting ROCK2 expression may be a strategy for cancer therapy.

In the present study, we found that ROCK2 mRNA and protein levels were reduced in PC cells following T4O treatment *in vitro*. High expression of ROCK2 was observed in PC tissues compared to adjacent tissues and indicated lower DFS and OS. ROCK2 overexpression markedly suppressed the effects of T4O on PC cell proliferation and mobility. Furthermore, *in vivo* assays showed that T4O had the potential to inhibit PC cell proliferation and decrease EMT biomarkers, KI67, PCNA and ROCK2 in tumor tissues. These results indicated that ROCK2 is involved in the T4O-induced biological processes.

## Conclusion

T4O treatment causes ROCK2 downregulation, thus leading to the inhibition of PC cell proliferation and migration.

## Supplementary Material

Supplemental Material

## Abbreviations

T4O, terpinen-4-ol; PC, pancreatic cancer; ROCK2, Rho-associated coiled-coil containing protein kinase 2; BCA, bicinchoninic acid; IHC, immunohistochemical staining.
