# Supplementary material for: Terpinen-4-ol inhibits the proliferation and mobility of pancreatic cancer cells by downregulating Rho-associated coiled-coil containing protein kinase 2
Source: Bioengineered. 2022 Mar 24;13(4):8643–56. doi: 10.1080/21655979.2022.2054205 (PMC9161900; doi:10.1080/21655979.2022.2054205)
Supplement: Supplemental Material [file KBIE_A_2054205_SM2271.docx]

| ID | RNA-seq (LogFC count+1) | qRT-PCR （relative expression in PANC-1） | qRT-PCR （relative expression in AsPC-1） |
| --- | --- | --- | --- |
| ARHGEF37 | 2.586** | 1.334* | 1.229* |
| ARHGEF6 | 1.503** | 1.003 | 0.989 |
| MYL9 | 1.521** | 1.322* | 1.443** |
| ARHGAP35 | -1.281* | 0.773* | 0.698* |
| ARHGEF11 | -1.114* | 0.744* | 0.711* |
| ARHGEF2 | -1.047* | 0.812* | 0.776* |
| ARHGEF28 | -1.863** | 0.662* | 0.499** |
| MYL10 | -1.040* | 0.934 | 0.898 |
| MYL2 | -2.162** | 0.668** | 0.521** |
| MYL5 | -1.036* | 0.942 | 0.988 |
| MYL7 | -1.000* | 0.832* | 0.722* |
| RAC3 | -1.614** | 0.769* | 0.712* |
| ROCK2 | -7.976** | 0.339** | 0.244** |
